# Measuring Non-Destructively the Total Indium Content and Its Lateral Distribution in Very Thin Single Layers or Quantum Dots Deposited onto Gallium Arsenide Substrates Using Energy-Dispersive X-ray Spectroscopy in a Scanning Electron Microscope

**DOI:** 10.3390/nano12132220

**Published:** 2022-06-28

**Authors:** Thomas Walther

**Affiliations:** Department of Electronic & Electrical Engineering, University of Sheffield, Mappin Building, Mappin Street, Sheffield S1 3JD, UK; t.walther@sheffield.ac.uk

**Keywords:** scanning electron microscopy (SEM), energy-dispersive X-ray spectroscopy (EDXS), InGaAs, Stranski–Krastanov, quantum dots

## Abstract

The epitaxial deposition of a precise number, or even fractions, of monolayers of indium (In)-rich semiconductors onto gallium arsenide (GaAs) substrates enables the creation of quantum dots based on InAs, InGaAs and indium phosphide (InP) for infrared light-emitting and laser diodes and the formation of indium antimonide (InSb)/GaAs strained layer superlattices. Here, a facile method based on energy-dispersive X-ray spectroscopy (EDXS) in a scanning electron microscope (SEM) is presented that allows the indium content of a single semiconductor layer deposited on a gallium arsenide substrate to be measured with relatively high accuracy (±0.7 monolayers). As the procedure works in top-down geometry, where any part of a wafer can be inspected, measuring the In content of the surface layer in one location without destroying it can also be used to map the lateral indium distribution during quantum dot formation and is a method suitable as an in-situ quality control tool for epitaxy.

## 1. Introduction

For InAs [1] and InGaAs [2] quantum dots and quantum wells deposited on GaAs substrates, the layer widths, quantum dot size and their relative indium content determine the optical properties of the quantum structures formed [3]. These parameters are all directly related to the total amount of indium deposited, which therefore needs to be measured reliably to control the deposition rate [4]. The Stranski–Krastanov transition from flat layer growth of homogenous In(Ga)As quantum wells to self-organised quantum dot formation occurs at a critical thickness that is itself temperature dependent [5] but is generally believed to lie around 1.7 monolayers (ML) of indium (In) [6] if a minimum indium concentration threshold is reached in the deposition above which indium segregation commences due to strain [7,8,9]. In-situ measurements such as intensity fluctuations in reflection high-energy electron diffraction (RHEED) [10] or spectroscopic ellipsometry (SE) [11] may be available during molecular beam epitaxy or low-pressure chemical vapour deposition, but sometimes the results are ambiguous when either the signals are blurred because the surfaces are not atomically flat or if some fraction of atoms deposited become re-evaporated at higher deposition temperatures. Ex-situ surface analysis techniques such as Auger electron spectroscopy [12], high-resolution electron energy-loss spectroscopy [13] or X-ray photo electron spectroscopy [14,15,16] can measure the total amount of In atoms deposited on a GaAs surface, if carefully calibrated, but need a transfer to another ultra-high vacuum chamber unless directly connected to the growth chamber, which is expensive and needs expert user operation. If just the total amount of indium on a surface were to be determined, micro-X-ray fluorescence (µ-XRF) would be an equally non-destructive measurement approach. It has been shown that ≈1% of a full monolayer of Sn could be detected on Si using this method [17], but the ability to subtract the bremsstrahlung background intensity accurately and reproducibly is critical, making the choice of the emission line, as well as the positions of substrate lines, important factors, so the sensitivity for In on GaAs will probably be much worse. Braun and Ploog [18] estimated the sensitivity for In on GaAs as 0.25 monolayers (ML) using a home-built kit within a growth chamber that provided time resolution but no spatial resolution, and at the growth temperature employed some indium that may already have started to desorb so that the measured In signal in the flux could have differed from the amount of In finally deposited. Generally, the lateral resolution of µ-XRF lies in the range >5 µm [19] and so would not allow simultaneous sub-µm distribution mapping of In-rich quantum dots.

Here, it is shown that transfer to a very basic table-top scanning electron microscope equipped with a tungsten hairpin emitter and an energy-dispersive X-ray spectrometer (SEM-EDXS) is a facile and relatively quick alternative characterisation method to measure sub-monolayer thicknesses of In-containing semiconductors on GaAs and to map the lateral distribution of In on the surface. Such equipment is now very widely spread and its availability in many teaching and research laboratories means that SEM-EDXS is rapidly becoming a routine technique, making it useful to consider signal quantification and explore its limitations.

In scanning electron microscopy, the energy of the primary electrons defines their interaction volume with the sample. This interaction volume increases both laterally and vertically with acceleration voltage, i.e., with electron energy [20]. The dependence of lateral resolution on the acceleration voltage has been studied extensively. The penetration depth of electrons into the sample also influences the depth in which the majority of X-rays are produced. This has been traditionally used in the past to adjust the depth sensitivity of SEM-EDXS and to measure the thicknesses of surface coatings, however, mainly for relatively thick layers in the range >0.05 µm, up to several µm [21,22,23].

Recent developments of dedicated instrumentation and modelling in wavelength-dispersive X-ray microanalysis (SEM-WDX) have shown that low-energy spectra taken with very bright electron beams under ultra-high vacuum conditions with multiple spectrometers can allow oxide thicknesses down to 10 nm [24] to be measured; a commercial publication claimed sensitivity down to 2 nm [25]. In a recent study, we have shown that thin oxide layers on top of Si and SiGe wafers could be measured in SEM-EDX top-down geometry to sub-nm accuracy [26]. In this study, the same principle is applied to the measurement of thin indium containing layers (InAs, InGaAs, InP, InSb) that are orders of magnitude thinner than typical hard coatings used for mechanical tools or anti-reflective coatings in optics; hence, X-ray count rates will be much lower and both lower acceleration voltages and the effects of external influences, such as the build-up of thin contamination layers of the surfaces during data acquisition, need to be considered in detail. Both our previous study [26] and our current study are examples of how basic SEM-EDXS instrumentation can, to some degree, substitute the use of much more complicated and expensive surface analysis equipment, which typically would have to operate in ultra-high vacuum conditions.

## 2. Monte Carlo Simulations

For modelling the electron penetration, multiple electron scattering, X-ray production and X-ray detection in SEM-EDXS [20,27] as a function of acceleration voltage the Monte Carlo package CASINO version 2.42 (Electron Microscopy Research Group, McGill University, Montreal, QC, Canada) has been used [28]. This code simulates the X-rays generated by multiply scattered electrons and those detected by a typical solid-state Si-based detector with an atmospheric thin polymer entrance window. It does, however, neglect secondary X-ray fluorescence effects, which seems to have only recently been considered in detail [29], and X-ray fluorescence from fast secondary electrons, which can be a problem at high acceleration voltages [30,31].

**Figure 1:**
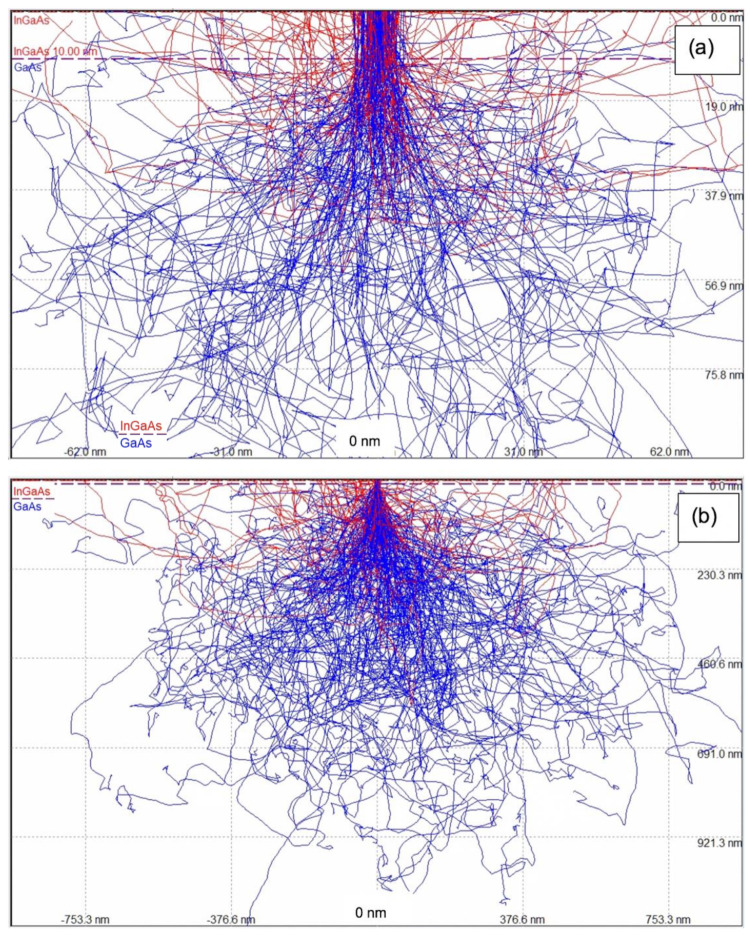
Monte Carlo simulation of multiple electron scattering for a 10 nm wide beam through 10 nm In_0.1_Ga_0.9_As film on GaAs substrate at energies of 4 keV (**a**) and 15 keV (**b**). Trajectories of back-scattered electrons are shown in red, those of absorbed electrons in blue. Note the different scales: (**a**): 155 nm × 94 nm, (**b**): 1883 nm × 1152 nm.

In order to obtain an unambiguous chemical signal from the indium (In) atoms in the surface layer, characteristic X-rays from the In L-line need to be excited. This line consists of the following prominent sub-lines: Lα_1_ at 3287 eV and Lα_2_ at 3279 eV (that cannot be separated by EDX spectrometers), Lβ_1_ at 3487 eV, Lβ_2_ at 3714 eV and Lγ_1_ at 3921 eV. So, the minimum electron energy needed for their excitation will be 4 keV. There are also two weaker sub-lines identified as L_l_ at 2904 eV and Lγ_2,3_ at 4160 eV. Figure 1 shows two screen shots from the graphical user interface of the CASINO software, one for electrons at 4 keV (top) and one for electrons at 15 keV (bottom), that demonstrate in this side-view that most electrons will penetrate rather deep into material, where they will be scattered multiple times and can produce X-rays from both the surface layer and the underlying substrate.

**Figure 2:**
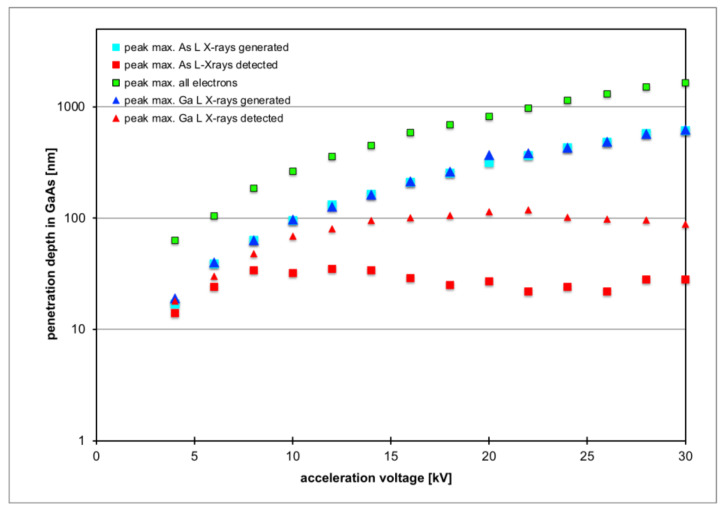
Monte Carlo simulation of the peak positions of the depth distributions of electrons (green squares), As L-line X-rays (turquoise and red squares) and Ga L-line X-rays (blue and red triangles) in GaAs as function of electron acceleration voltage.

Figure 2 quantifies this by plotting from the depth profiles for each energy the maximum peak position of the corresponding depth distribution of electrons and X-rays for the range of 4–30 keV electron energy. The simulated electron penetration depth increases from 63 nm at 4 kV to 1640 nm at 30 kV, while the depth distributions of X-rays *generated* from the As and Ga L-lines increase from 18 nm at 4 kV to 620 nm at 30 kV; however, the peak depth from which most of these X-rays are actually *detected* is much shallower because many soft X-rays from larger depths will be re-absorbed in the sample. In particular, As L-line X-rays persistently come from a depth of up to only ≈30 nm and can therefore still be regarded to originate relatively close to the surface. Hence, As L will constitute an ideal calibration signal with which to compare the In L-line intensity.

**Figure 3:**
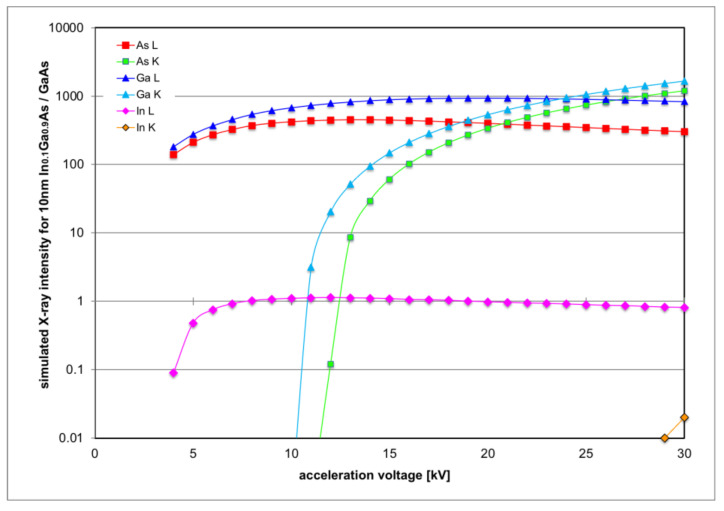
Monte Carlo simulation of the intensities of various X-ray lines for 10 nm In_0.1_Ga_0.9_As/GaAs as function of electron acceleration voltage.

Figure 3 shows X-ray intensities of all X-ray lines expected from (In)GaAs for 10 nm In_0.1_Ga_0.9_As/GaAs as a function of electron acceleration voltage. While the curves for the hard X-ray K-lines commence only above 11 keV and then increase rapidly, the curves for all L-lines are much smoother and reach peak values for acceleration voltages of 12 kV (In L), 14 kV (As L) and 20 kV (Ga L), so this range should generate optimal count rates.

**Figure 4:**
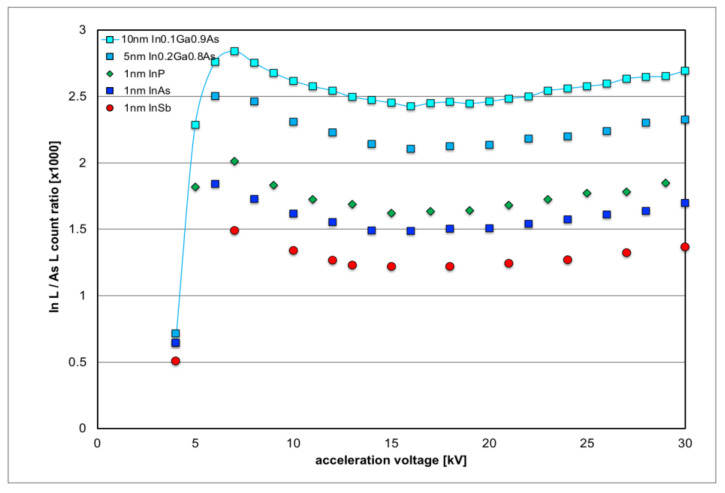
Monte Carlo simulation of the intensity ratio of In L/As L X-ray lines for various In-containing thin layers on GaAs as function of electron acceleration voltage.

In particular, the purple curve for In L-line intensity and the red curve for As L line intensity in Figure 3 are similar in form so that their ratio, plotted in Figure 4, should remain almost constant. In the following, a number of different thin overlayers have been simulated, as listed in Table 1, and the resulting In L/As L line intensity ratios are plotted as a function of acceleration voltage in Figure 4. While this intensity ratio does not stay constant, all curves are of a similar form, with an initial sharp increase from 4 kV to 5–6 kV and a slight and broad dip around 15 kV.

All layers considered here effectively contain a total indium amount corresponding to 1 nm InZ (where Z = P, As or Sb is a group-V element from the periodic table); however, due to the different lattice parameters, those layers with increased lattice parameters contain fewer In atoms per unit volume and, thus, also per unit area for such a 1 nm thick layer modelled. Hence, the curves in Figure 4 are all arranged in sequence of their lattice constants (increasing from top to bottom) or areal density of In atoms (decreasing from top to bottom). Any quantification by SEM-EDXS will be ultimately limited by the lack of knowledge of the precise strain state of the top surface layer that contains In atoms: relaxation modifies the form of its unit cells from compressively strained tetragonal with in-plane lattice constants identical to *a*_GaAs_ but a vertically increased lattice constant of *c* where [32]

(1) $$c=a_{\mathrm{GaAs}}+x\left(a_{{\mathrm{In}}_{x}{\mathrm{Ga}}_{1-x}Z}-a_{\mathrm{GaAs}}\right)\frac{1+\nu }{1-\nu }$$

with a Poisson number of $\nu \approx \nu_{\mathrm{GaAs}}=0.312,$ to that of a cubic sample *a*_In*x*Ga1–*x*Z_ in the relaxed case. So, while for the relaxed state the cubic unit cell with 4*x* In atoms has a volume of (*a*_In*x*Ga1–*x*Z_)^3^; for the strained state this tetragonal unit cell volume is given as *ca*_GaAs_^2^. Normalizing the data in Figure 4 not with respect to a 1 nm thin In-enriched overlayer but to the areal density of In atoms contained within this overlayer gives the values of the two last columns in Table 1. These values decrease from the GaAs value the more In atoms replace Ga atoms and the larger the corresponding group-V atom becomes, in line with the curves in Figure 4, which demonstrates that the In L/As L ratio can be used as a direct measure of the number of In atoms in the surface layer, within 7% maximum error for the In(Ga)As system.

**Figure 5:**
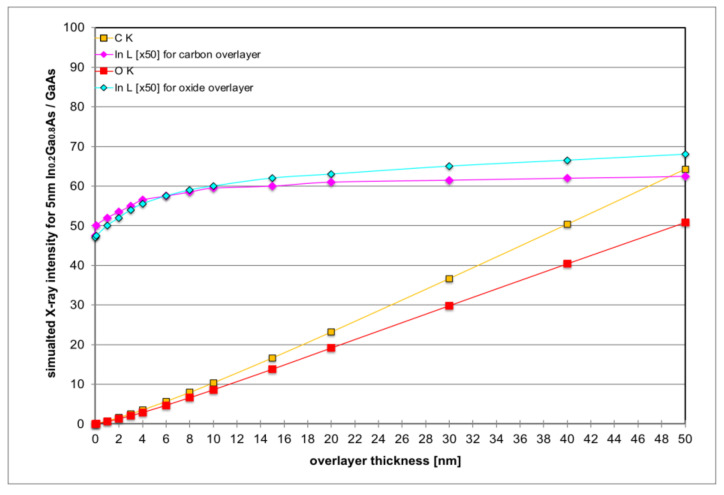
Monte Carlo simulation of the intensities of selected X-ray lines for 5 nm In_0.2_Ga_0.8_As on GaAs as function of overlayer thickness where the surface overlayers are modelled as pure carbon (amber squares for C K, purple diamonds for In L-line, × 50) or pure b-Ga_2_O_3_ (red squares for O K-line, turquoise diamonds for In L-line, × 50).

Figure 5 proves that very thin surface layers of native oxides or hydrocarbon contamination will not significantly influence the In L signal from the indium containing top semiconductor layer (here: 5 nm of In_0.2_Ga_0.8_As) as long as these surface layers remain sufficiently thin; a less than 10% relative increase in In L can be predicted for surface layers thinner than 3 nm, which can be guaranteed in practice as long as very high magnifications are avoided, otherwise surfaces may need to be plasma cleaned prior to analysis [33].

## 3. SEM Characterisation

Comparable spectroscopy experiments were performed under the same conditions by mounting several specimens onto a common stub for investigation in a Hitachi table-top SEM 3030 plus (Hitachi High-Tech, Krefeld-Fichtenhain, Germany) operated at *U* = 15 kV, a take-off angle (TOA) of 22° and magnification of 40 ×. The microscope is equipped for EDXS with a 30 mm^2^ Bruker XFlash 430 (Bruker Nano GmbH, Berlin, Germany) silicon drift detector of nominal 450 µm thickness, which has a 0.029 mm dead layer, an AP3.3 polymer entrance window and provides 126 eV full width at half maximum resolution at 5.9 keV and a sampling of 10 eV/channel. The surfaces of all specimens were cleaned in 99.8% pure ethanol and acetone. No conductive coatings were applied to any sample.

**Figure 6:**
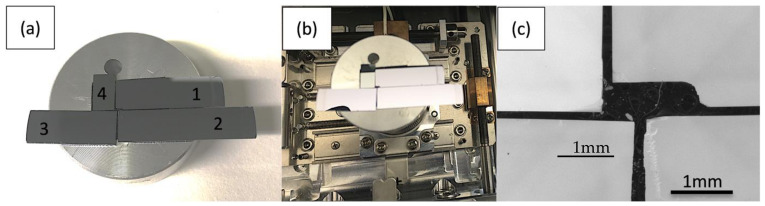
Three long specimens of InGaAs/GaAs (numbers # 1–3) and one small piece of InAs substrate (sample # 4) mounted on a 1” diameter SEM stub (**a**,**b**); BSE image of central area where all samples are visible, as used for SEM-EDXS (**c**). Specimen numbers correlate with those in Table 3.

Figure 6 shows four of the six specimens examined in this study (the ones not shown here are Si and GaAs wafers). Three are thin In_x_Ga_1-x_As single layers of nominal thicknesses of 2, 5 and 10 nm, respectively, grown on GaAs substrates by molecular beam epitaxy. Their indium content *x* = 0.20 is believed to be calibrated within ∆*x* = 0.01, as were those samples in references [7,8,9]. The fourth sample is a small piece from an S-doped InAs wafer. Note the slight difference in hue of the InAs and (In)GaAs surfaces in the optical image of Figure 6b due to the different bandgaps of the materials. As the bandgap decreases, the reflectivity peaks of InGaAs shift from the blue to the green-yellow region [34,35]. Figure 6c is an SEM image taken with the back-scattered electron (BSE) detector, recorded at 15 kV and 40 × magnification with a working distance of 7.7 mm. Here, the InAs wafer surface appears 20–30% brighter than those of (In)GaAs, while simulations (cf. Table 2) only suggest a relative increase in the BSE yield from GaAs to InAs of ≈10%. The difference may be explained by the weak channelling contrast of the (001) oriented wafer pieces, visible as faint dark contours running along horizontal, vertical and diagonal directions in Figure 6c.

### 3.1. Energy-Dispersive X-ray (EDX) Spectroscopy

**Figure 7:**
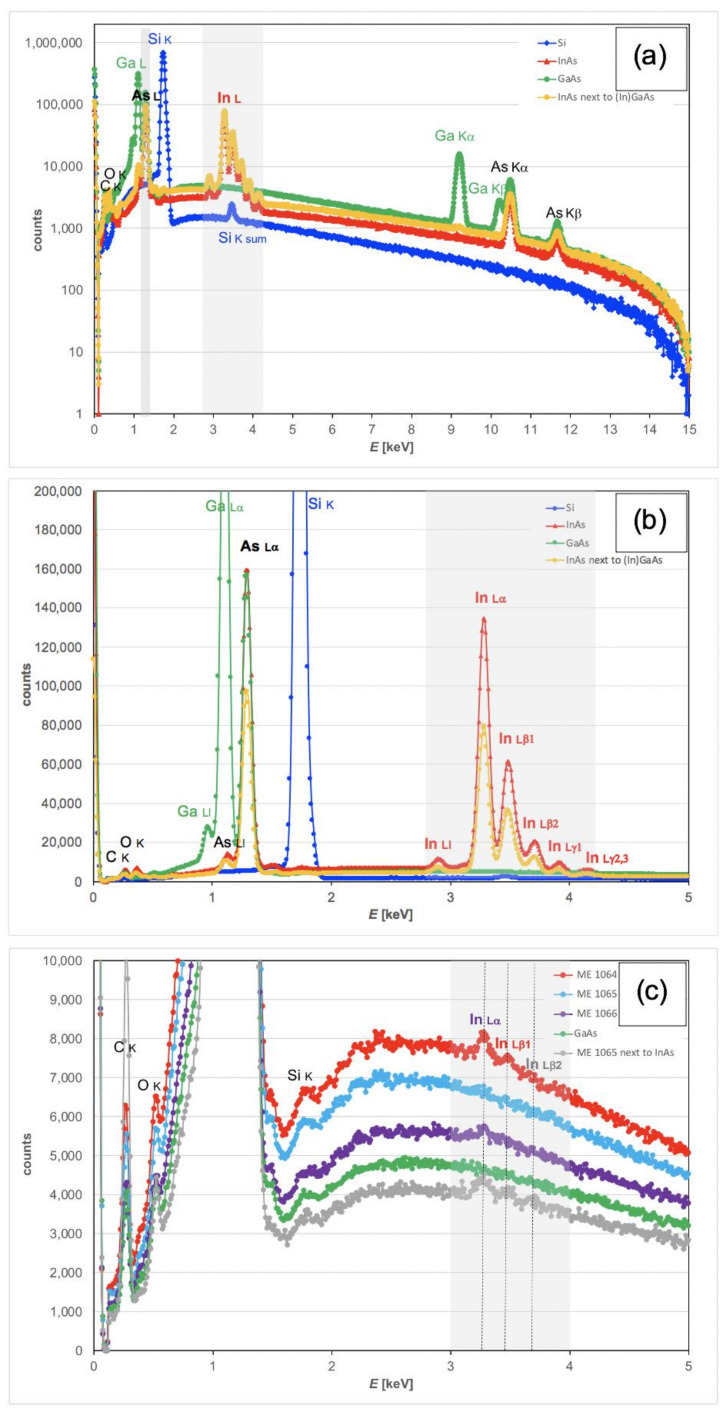
X-ray spectra from InAs, GaAs, Si and three InGaAs/GaAs wafer samples, displayed on a logarithmic scale for the energy range of 0–15 keV (**a**), on linear scale for the relevant range of 0–5 keV only, using different vertical scales to show the strong In L and As L lines (**b**) and the weak In L signals in the epitaxial wafer samples (**c**).

For the spectra recorded with acquisition times of 1–1.15 h as shown in Figure 7, wafers were investigated pairwise, as it turned out that the weak In signal from the InGaAs layer samples would be influenced by stray scattering from InAs if simultaneously present, as shown here.

The software Quantax 70 (Bruker Nano GmbH, Berlin) automatically quantifies characteristic X-ray lines tabulated for elements from Be (*Z* = 4) to Cf (*Z* = 98) in four steps:

i. It removes detector artefacts, such as sum peaks, escape peaks, offsets due to incomplete charge collection and blur due to line broadening.
ii. It performs continuous bremsstrahlung fitting and background subtraction around these characteristic lines [36].
iii. It fits the peaks of all characteristic X-ray lines detected, including multiple sub-lines.
iv. It quantifies the net counts extracted by internal, standardless calculation of element-number (*Z*) specific ionisation cross-sections, fluorescence yields and relative X-ray emission rates (*Z*-effect), absorption (*A*) and internal fluorescence (*F*) corrections. This is called standardless peak-to-background (*P/B*) *ZAF* quantification [37,38].

### 3.2. EDX Mapping

**Figure 8:**
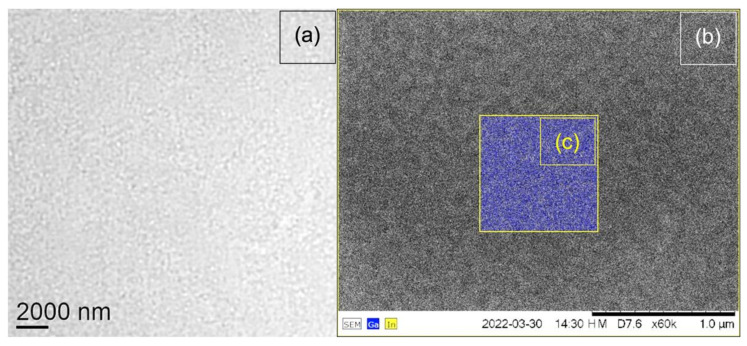
Optical light micrograph of (20 µm)^2^ surface area in reflection (**a**) and BSE image (**b**) with X-ray map insert of (0.9 µm)^2^ area (**c**) with Ga K counts in blue (min = 41, max = 209) and In L counts in yellow (min = 0, max = 5). All bright dots in (**c**) are yellow and, despite the noise, align well with the white dot pattern in (**b**).

X-ray mapping was conducted for sample # 6 of nominally 1.5 ML InAs on a GaAs substrate, which showed 200–300 nm scale speckle-type contrast in light microscopy, as shown in Figure 8a. These features are of the same dimension as the nominal 210 nm resolution of the light microscope used. Hence, the direct interpretation of this speckle from light microscopy is not possible. A back-scattered electron image (Figure 8b), however, indicates this speckle pattern is a real feature of the surface, and the small area in its centre investigated by X-ray mapping (Figure 8c) confirms the presence of a rough surface where In-rich islands and In-poor troughs and holes form a meandering surface pattern typical of surfaces just before the Stranski–Krastanov transition from flat layer to island growth, via the percolation and roughening of the wetting layer already formed.

## 4. Discussion

Let us first look at X-ray spectra of the pure binary materials InAs and GaAs in Figure 7. InAs shows an In_L_/As_L_ ratio of 1.60 in the experimental spectra (yellow-orange), while Monte Carlo simulations would have suggested a ratio of 1.44 (see Table 2). This 10% relative discrepancy is small, given that the SDD detector used in the experiment is somewhat thinner than the Si:Li detector used in the simulations, and it is not quite clear which of the In L sub-lines are actually taken into account in the model simulation. In any case, this error in the effective *k*-factors is negligible compared to the scatter due to the uncertainty in the distribution of indium in various top layers (InGaAs, InAs, InP or InAs, cf. Figure 4). The X-ray spectrum of GaAs (green) shows no apparent In signal but the quantitative analysis by the Bruker Quantax software reports about 600 net counts for In L, with a statistical error bar of ±670 counts based on integrating, on average, about 4500 counts over 100 channels (from 3–4 keV). This suggests a baseline for In_L_/As_L_ ratio detectability of (0.4 ± 0.5)‰, or 400 ppm. This is almost as large as the signal expected from 1 ML pure InAs and indicates this will be the detection limit in our set-up for measurements about 1 h long, where the specimen covers half the field of view (as two specimens were always studied in parallel); an improvement would only be possible by reducing the noise visible in the spectra of Figure 7c by increasing the beam current of the tungsten filament, simply looking at one wafer surface only, or by increasing the measurement time correspondingly; an improvement in the SNR by a factor of 4 would be possible in this instrument if one wafer surface was monitored for a whole 8 h working day.

For wafer ME1064 in Figure 7c, one can clearly see the peaks of two In L-lines (Lα and Lβ1), for wafer ME1066, only the strongest line (Lα) remains visible, and for wafer ME1065, the human eye can no longer discern a clear peak in the range of 3–4 keV, in agreement with the software reporting net counts of around 1100, which is close to the upper limit of the zero baseline signal of 600 ± 800 counts for GaAs. The count rates, In_L_/As_L_ ratios and extracted equivalent In full monolayers are all listed in Table 3.

Figure 8 demonstrates the feasibility of mapping the lateral indium distribution on a surface by SEM-EDX. It should be noted that the In/As ratio from EDXS of this specimen is almost a factor 2 higher than expected, possibly due to some underlying InGaAs containing a buffer layer that the 15 kV electron beam partially penetrates. Furthermore, the In/Ga ratio from the X-ray maps in Figure 8c is much higher than expected from the spectrum, by a factor of 3–5, which is attributed to bit-rounding errors when generating non-negative integer distribution maps: the Ga map should have had a mean of only 0.2 counts (i.e., consisting mainly of pixels with 0 and 1 counts) but actually had a mean of almost unity. This indicates that full quantification of SEM-EDXS maps with low count rates may not be possible with standard commercial software, similar to that found for STEM-EDXS [39]. Still, the observed speckle patterns in Figure 8a,b agree in length scale.

A direct correlation of these SEM-EDXS results with surface analytical techniques mentioned previously has so far not been possible for several reasons: AES would need to carefully calibrate the escape depths of Auger electrons for InGaAs, which is beyond the scope of this study; SIMS does not provide reliable signals from the top surface monolayer, as ion currents typically need some seconds to stabilise [40]; and µ-XRF is not normally used for low X-ray energies such as As L or even In L-lines. There is, to our knowledge, only one recent example of µ-XRF incorporated into SEM-EDXS (called *XTrace*, by Bruker) that could potentially be used to compare the two techniques; however, no such instrument seems to be available in the UK. The manufacturer claims it will work well for thin films above 1 nm thickness and achieve down to 10 ppm sensitivity for homogeneous glass samples. From its specification (50 kV anode voltage, 600µA anode current [41]), it is clear the penetration of 50 kV X-rays into the sample will be almost one order of magnitude larger than that of 15 keV electrons used here, yielding much lower X-ray counts from the surface for similar beam currents, but then X-ray anodes can be run at typically one order of magnitude higher currents than tungsten hairpin filaments (≈50 µA), which would compensate the lower surface sensitivity. While the background in µ-XRF is much lower than in EDXS, the major advantage would be that an inherent consistency check could be performed by comparing µ-XRF results from In_L_/As_L_ ratio quantification with In_K_/As_K_ ratios using MC simulations similar to those shown here. This is planned for a future study.

Finally, it should be noted that in principle, a tilt of the sample towards the X-ray detector could be used to enhance the surface sensitivity of the indium signal due to grazing incidence; however, very large angles would be required (84.3° for a factor of 10×, 89.43° for a factor of 100×) and a reliable, nearly vertical set-up would be extremely difficult to achieve in practice. Many basic SEMs, such as the model used here, do not have any sample tilt stages and the only option would be to mount the samples near-vertically on a suitable wedge, which, again, cannot be achieved with sub-degree precision.

## 5. Conclusions

The measurements indicate that just under 1 ML equivalent of In atoms (i.e., half a unit cell of InP, InAs or InSb) on GaAs is detectable in SEM-EDXS, as long as neither any Si wafer material nor any material with a high indium content, such as InAs wafers, are adjacent to the region of analysis: a strong Si signal would yield a small sum peak at 3.48 keV (cf Figure 7a,b) that could be misinterpreted by the software as being due to In Lβ_1_. Additionally, an InAs wafer in the vicinity, as shown in Figure 6a, would give a false positive signal for (In)GaAs (cf. grey curve in Figure 7c) due to In L-line stray electrons that can extend laterally over distances of several millimetres [29,30,31].

A higher electron dose (longer exposure and/or higher beam current) would improve the signal-to-noise ratio further so that In_L_/As_L_ ratios <400 ppm may be detectable, as long as no additional peaks appear in the 2.5–4.5 keV range that would change background fitting and subtraction. The above value is similar to the minimum detectable mass limit estimated for EDXS in [42] and corresponds to 0.65 ML, or 4 atoms/nm^2^. In our Hitachi 3030 plus table-top SEM, we typically obtained for GaAs samples about 2 million net counts of the As L edge within 1–1.15 h of acquisition. The detection limit scales with 1/√*t*, where *t* is the acquisition time. Error bars under ± 0.1 ML, which have been obtained for buried InGaAs layers by TEM-EDX in cross-section geometry [39,43,44,45] and by SEM-WDX for sulphur [46] will probably not be obtainable by SEM-EDX in plan-view geometry because this would necessitate about 100 million net counts for the As L edge, which, on this table-top SEM, would take 50–60 hrs of data acquisition and is unrealistically long for quality control. SEM instruments with field-emission sources that generate higher beam currents and larger X-ray detectors that provide higher collection angles may be able to approach this limit, which, of course, would be highly desirable for quality control purposes.

## Figures and Tables

**Table 1:** Materials properties used in simulations and volume density of indium atoms for various overlayer materials considered. Note that materials with a larger lattice constant have fewer In atoms in an equivalent layer of 1 nm thickness.

Layer Material	Density	Band-	Lattice		# In Atoms/nm^3^	
	[g/cm^3^]	gap [eV]	Constant [pm]	Strain State Compared	Relaxed	Fully Strained
		@300K	@300K	to GaAs		
GaAs	5.32	1.42	565.3		22.14	–
In_0.1_Ga_0.9_As	5.34	1.27	569.4		21.67	21.84
In_0.2_Ga_0.8_As	5.38	1.13	573.4		21.21	21.55
InP	4.81	1.344	586.87		19.79	20.64
InAs	5.68	0.354	605.83		17.99	19.48
InSb	5.77	0.17	647.9		14.71	17.32

**Table 2:** Monte Carlo simulation of backscattered electron (BSE) yield and characteristic X-ray intensities of L- and K- lines for bulk substrates at 15 kV.

Bulk Sample		BSE Coeff.	As X-rays		Ga X-rays		In X-rays	
	Line Type		L	K	L	K	L	K
GaAs		0.329	444.93	60.95	897.14	148.88	-	-
InAs		0.361	419.02	50.79	-	-	603.52	0

**Table 3:** List of specimens investigated and X-ray counts extracted for acquisition times of 1–1.15 h. Error bars are based on simple counting statistics for the background. The nominal InAs equivalent monolayers (ML) are the product of nominal layer thickness and nominal indium concentration of the group III sub-lattice, divided by half the relaxed unit cell thickness (*a*/2) which corresponds to the thickness of a (002) lattice plane.

#	Growth Number	Substrate	Top Layer	Nom. InAs Equiv. ML	In_L_ [Counts]	As_L_ [Counts]	In_L_/As_L_	Measured Thickness [InAs Equiv. ML]
1	ME1065	GaAs	In_0.2_Ga_0.8_As	1.4	1112	2,106,681	0.000528	0.9 ± 0.6
2	ME1066	GaAs	In_0.2_Ga_0.8_As	3.5	5239	1,696,135	0.003083	5.1 ± 0.7
3	ME1064	GaAs	In_0.2_Ga_0.8_As	7.0	12,380	2,392,187	0.005175	8.6 ± 0.6
4	–	InAs	–	–	2,342,596	1,463,727	1.60	–
5	–	GaAs	–	0	596	1,459,337	0.000408	0.7 ± 0.8
6	AF9151	GaAs	InAs	1.5	3787	2,279,822	0.001661	2.7 ± 0.8

## Data Availability

Not applicable.
